# Supplementary material for: Mapping the nucleolar proteome reveals a spatiotemporal organization related to intrinsic protein disorder
Source: Mol Syst Biol. 2020 Aug 3;16(8):e9469. doi: 10.15252/msb.20209469 (PMC7397901; doi:10.15252/msb.20209469)
Supplement: Supplementary file 1 — Appendix [file MSB-16-e9469-s001.pdf]

Appendix

Mapping the nucleolar proteome reveals a spatiotemporal organization related to intrinsic protein disorder

Lovisa Stenström[1], Diana Mahdessian[1], Christian Gnann[1,2], Anthony J. Cesnik[2,4], Wei Ouyang[1], Manuel D. Leonetti[2], Mathias Uhlén[1], Sara Cuylen-Häring[3], Peter J. Thul[1], Emma Lundberg[1,2,4*]

[1]Science for Life Laboratory, School of Engineering Sciences in Chemistry, Biotechnology and Health, KTH Royal Institute of Technology, SE-171 21 Stockholm, Sweden.
[2]Chan Zuckerberg Biohub, San Francisco, CA 94158, USA.
[3] Cell Biology and Biophysics Unit, Structural and Computational Biology Unit, European Molecular Biology Laboratory, Heidelberg, Germany.
[4]Department of Genetics, Stanford University, Stanford, CA 94158, USA.

[*]Correspondence to: emma.lundberg@scilifelab.se

Table of Contents:

Appendix Figure S1 Page 2
Appendix Figure S2 Page 3
Appendix Figure S3 Page 4
Appendix Figure S4 Page 5
Appendix Figure S5 Page 6
Appendix Figure S6 Page 7
Appendix Figure S7 Page 8
Appendix Figure S8 Page 9
Appendix Figure S9 Page 10
Appendix Figure S10 Page 11
Appendix Figure S11 Page 12
Appendix Figure S12 Page 13
Appendix Figure S13 Page 15

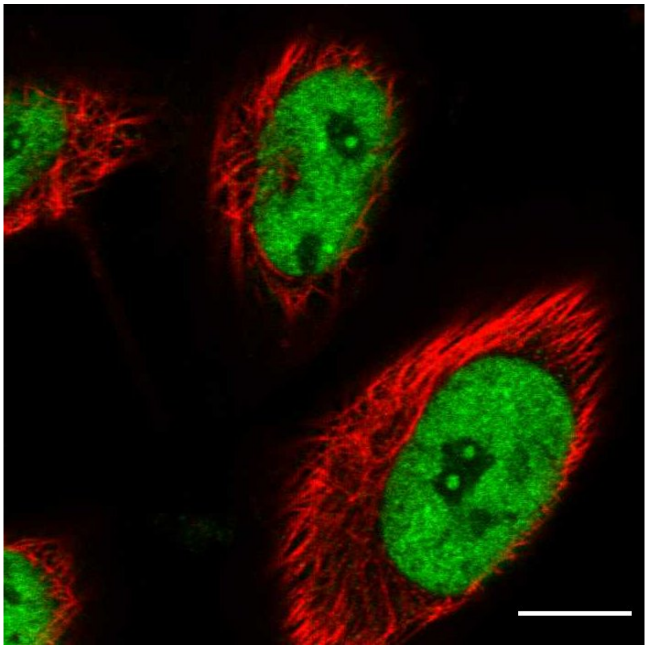

Appendix Figure S1

Immunofluorescent staining of LEO1 in HeLa wild type cells. Protein of interest is shown in green and microtubules in red. Scale bar 10 µm.

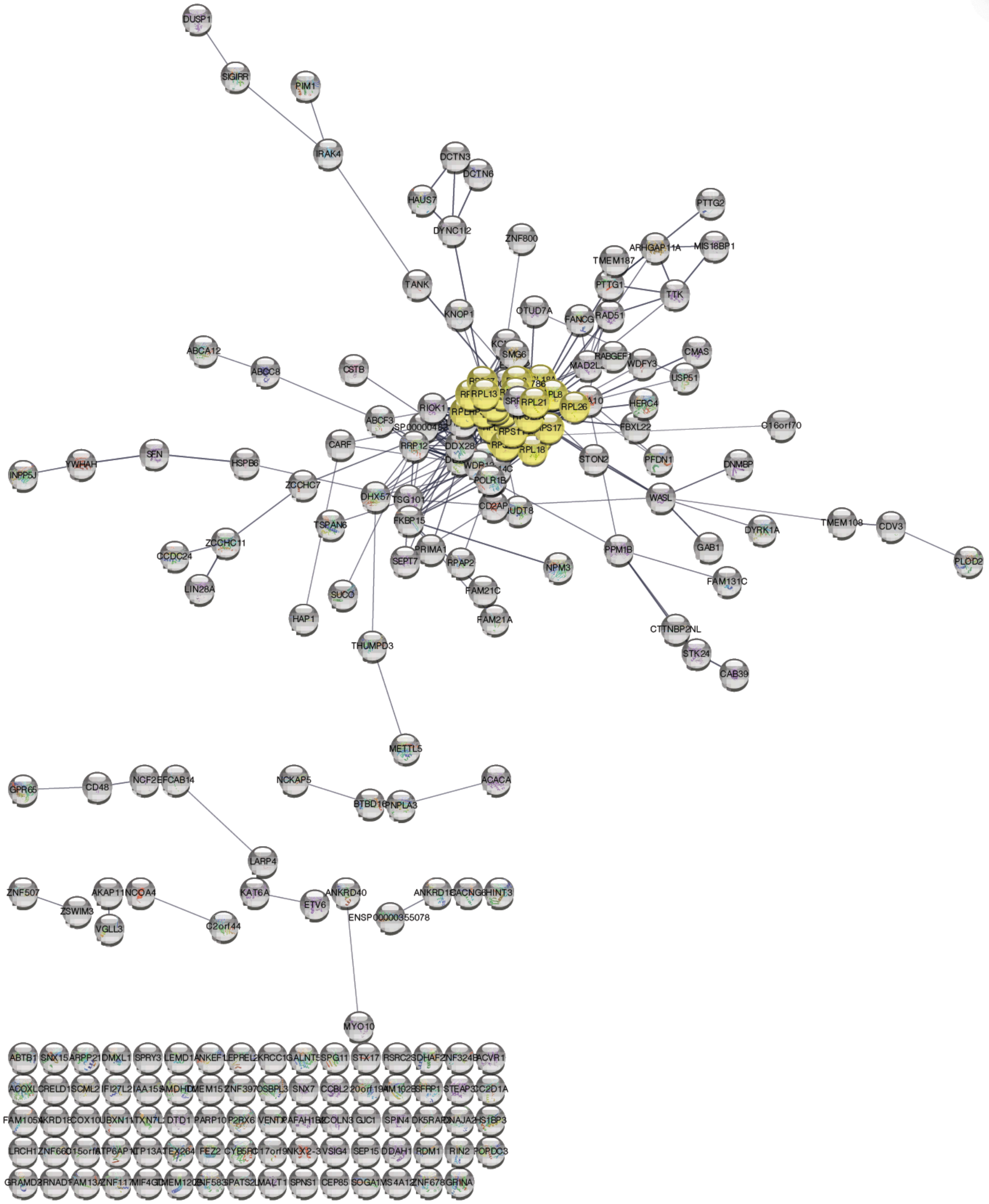

Appendix Figure S2

Protein-protein association network of the shared nucleolar and cytosolic proteome. The yellow highlighted nodes show ribosomal proteins as annotated in Gene Ontology.

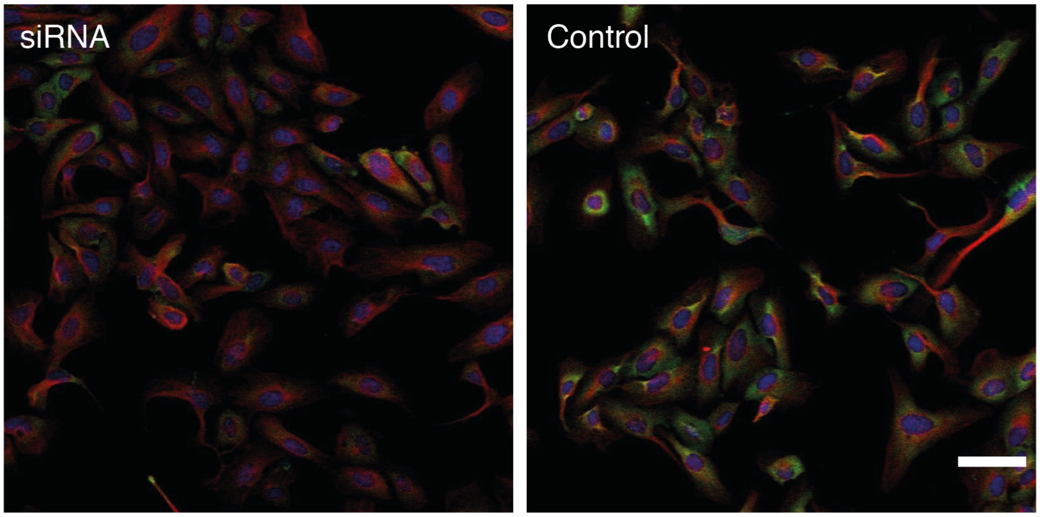

Appendix Figure S3

siRNA antibody validation data for RPL13 (HPA051702). siRNA treated cells to the left, control cells to the right. Protein of interest is shown in green, microtubules in red and DAPI in blue. Scale bar 20 μm.

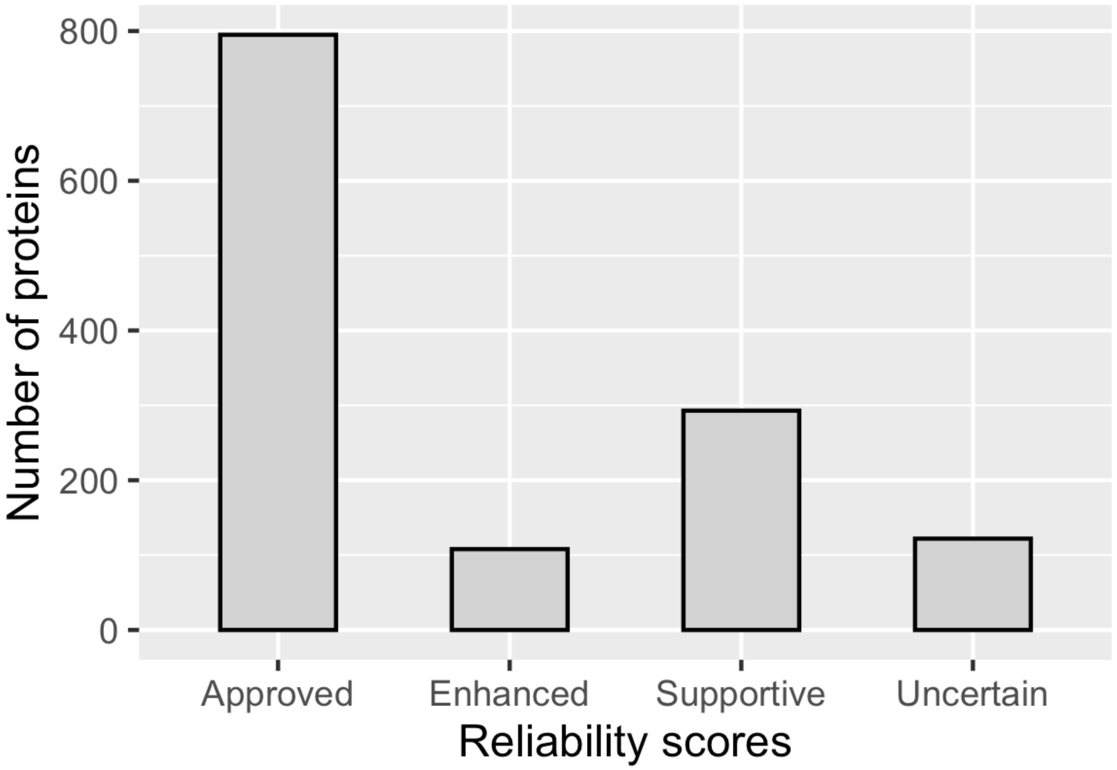

Appendix Figure S4

Distribution of the localization reliability scores for the nucleolar proteins in the HPA Cell Atlas (v.19), $n_{approved}$ = 795, $n_{enhanced}$ = 108 , $n_{supportive}$ = 293, $n_{uncertain}$ = 122.

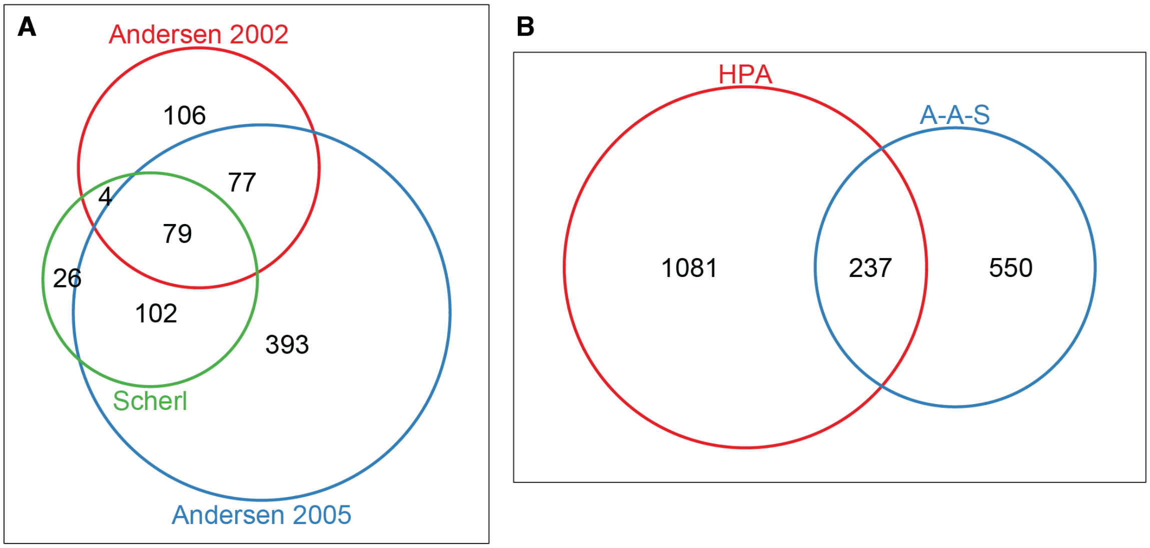

Appendix Figure S5

A. Overlap between the nucleolar proteins detected in Scherl *et al.* 2002, Andersen *et al.* 2002 and Andersen *et al.* 2005.

B. Overlap between the merged dataset from the studies mentioned above (A-A-S) and the nucleolar proteins in the HPA Cell Atlas (v19).

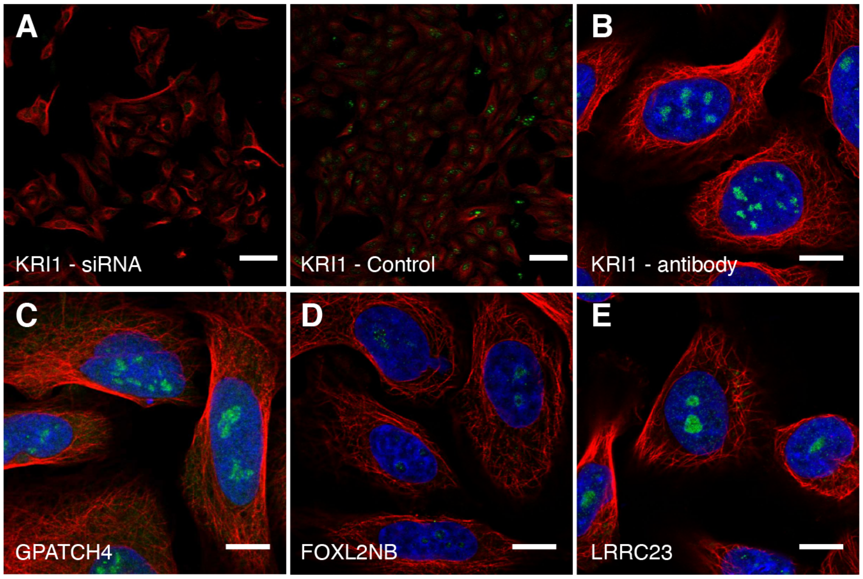

Appendix Figure S6

A. siRNA antibody validation data for KRI1 (HPA043574). siRNA treated cells to the left, control cells to the right. Scale bar 20 µm.

IF stainings of B. KRI1 (HPA043110), C. GPATCH4 (HPA028323), D. FOXL2NB (HPA071790) and E. LRRC23 (HPA037766) using antibodies targeting different epitopes of the protein, showing the same localization pattern as the original antibody. Protein of interest is shown in green, microtubules in red and DAPI in blue. Scale bar 10 µm.

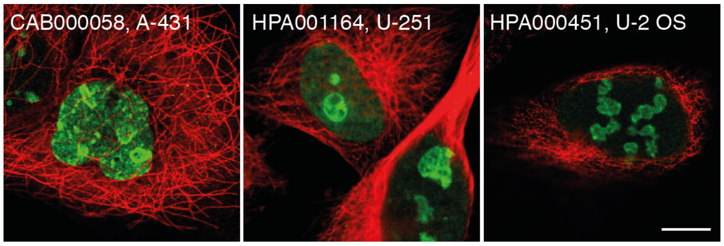

Appendix Figure S7

IF stainings of MKI67 using three antibodies targeting different epitopes of the protein. All showing the characteristic nucleoli rim staining pattern. Protein of interest is shown in green and microtubules in red. Scale bar 10 µm.

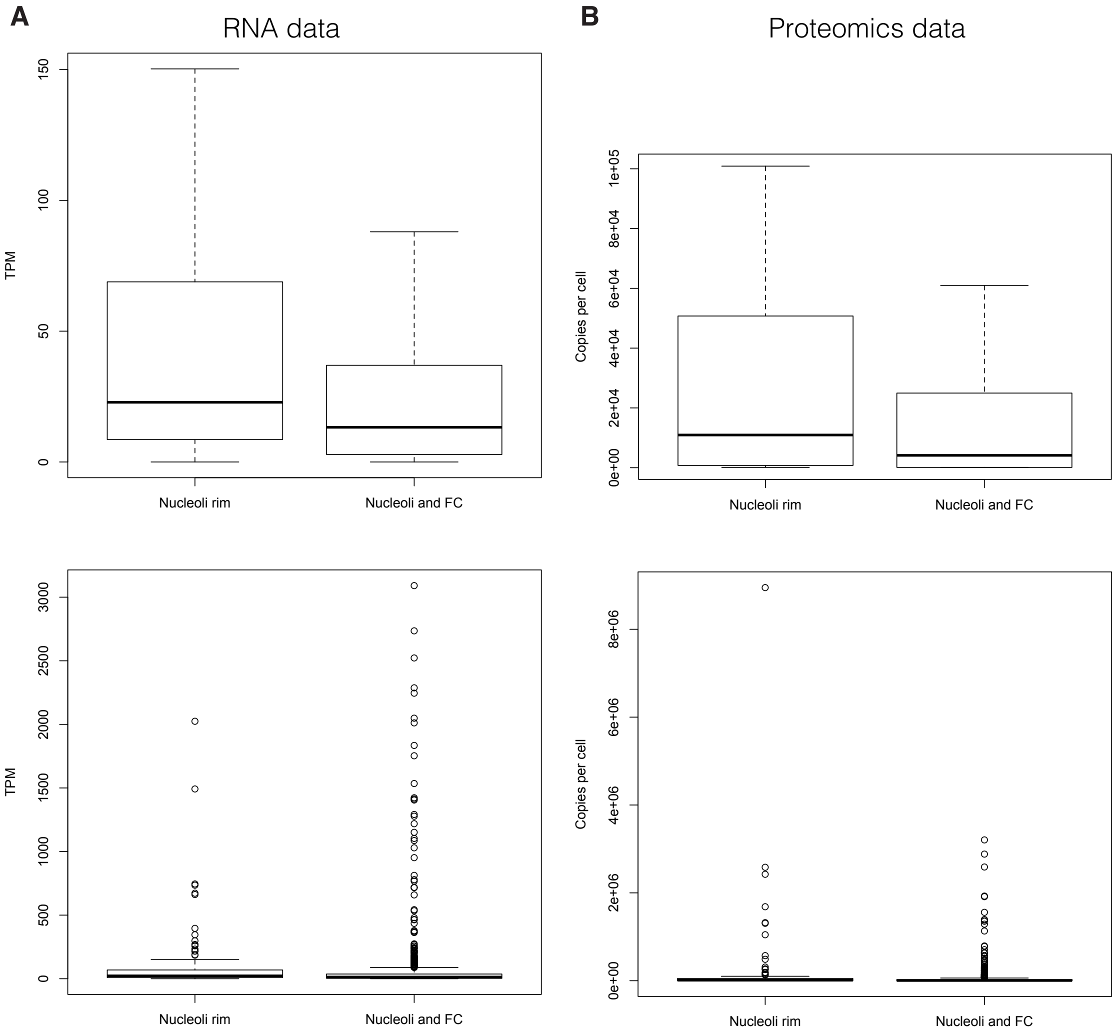

Appendix Figure S8

A. U-2 OS RNA expression data (TPM) and B. proteomics data (Copies per cell) from Beck *et al.*, 2011 for the nucleoli rim proteins compared to all other nucleolar proteins. In the proteomics dataset, 96 of 157 (61%) nucleoli rim proteins and 613 of 1,161 (53%) non-rim nucleolar proteins were detected. The upper box plots show the distribution of quartile one to four, while the lower box plots show the distribution of all genes/proteins in the dataset.

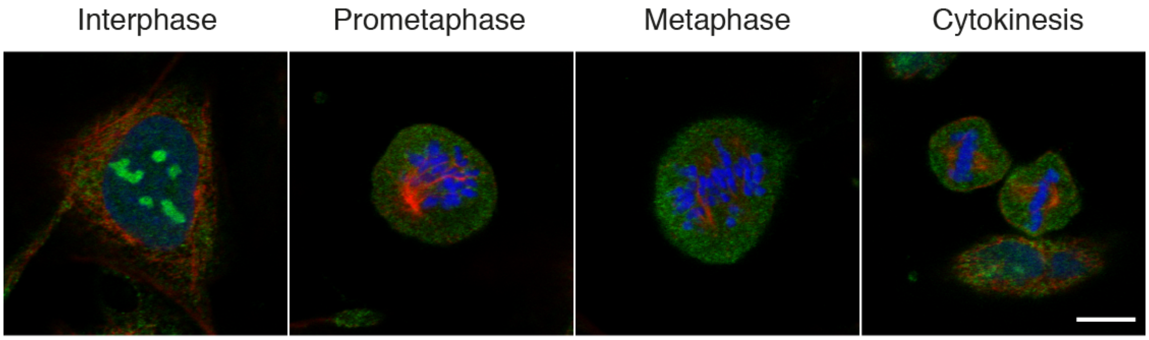

Appendix Figure S9

85 of 150 nucleolar proteins did not localize to mitotic chromosomes. Here exemplified by RPS19BP1 (HPA042874). Protein is shown in green, DAPI and blue and microtubules in red. Scale bar 10 μm.

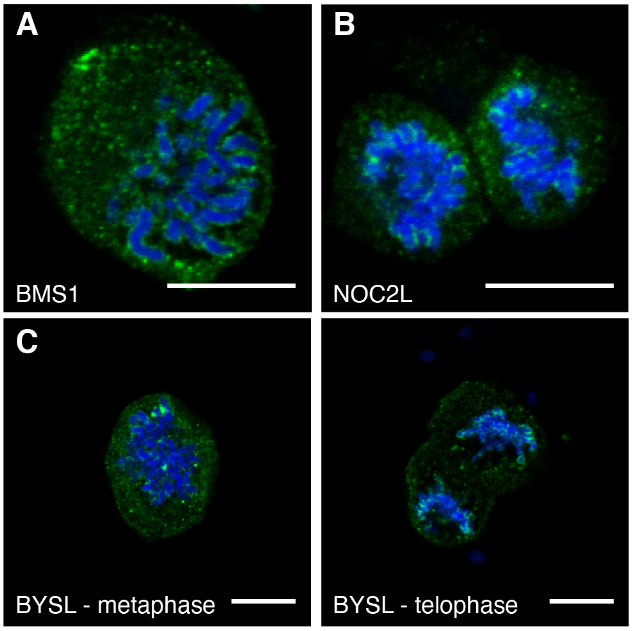

Appendix Figure S10

IF stainings of A. BMS1 (HPA036589), B. NOC2L (HPA062195) and C. BYSL (HPA031217) in mitosis using antibodies targeting different epitopes of the protein, showing the same localization on mitotic chromosomes as the original antibody. Protein of interest is shown in green and DAPI in blue. Scale bar 10 µm.

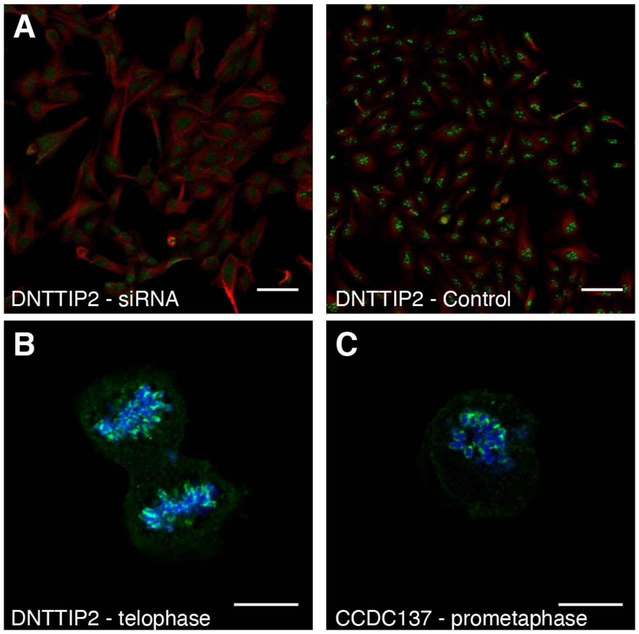

Appendix Figure S11

A. siRNA antibody validation data for DNTTIP2 (HPA044502). siRNA treated cells to the left, control cells to the right. Scale bar 20 µm.

IF stainings of B. DNTTIP2 (HPA044502) and C. CCDC137 (HPA053914) in mitotic cells showing localization to mitotic chromosomes. Protein of interest is shown in green and DAPI in blue. Scale bar 10 µm.

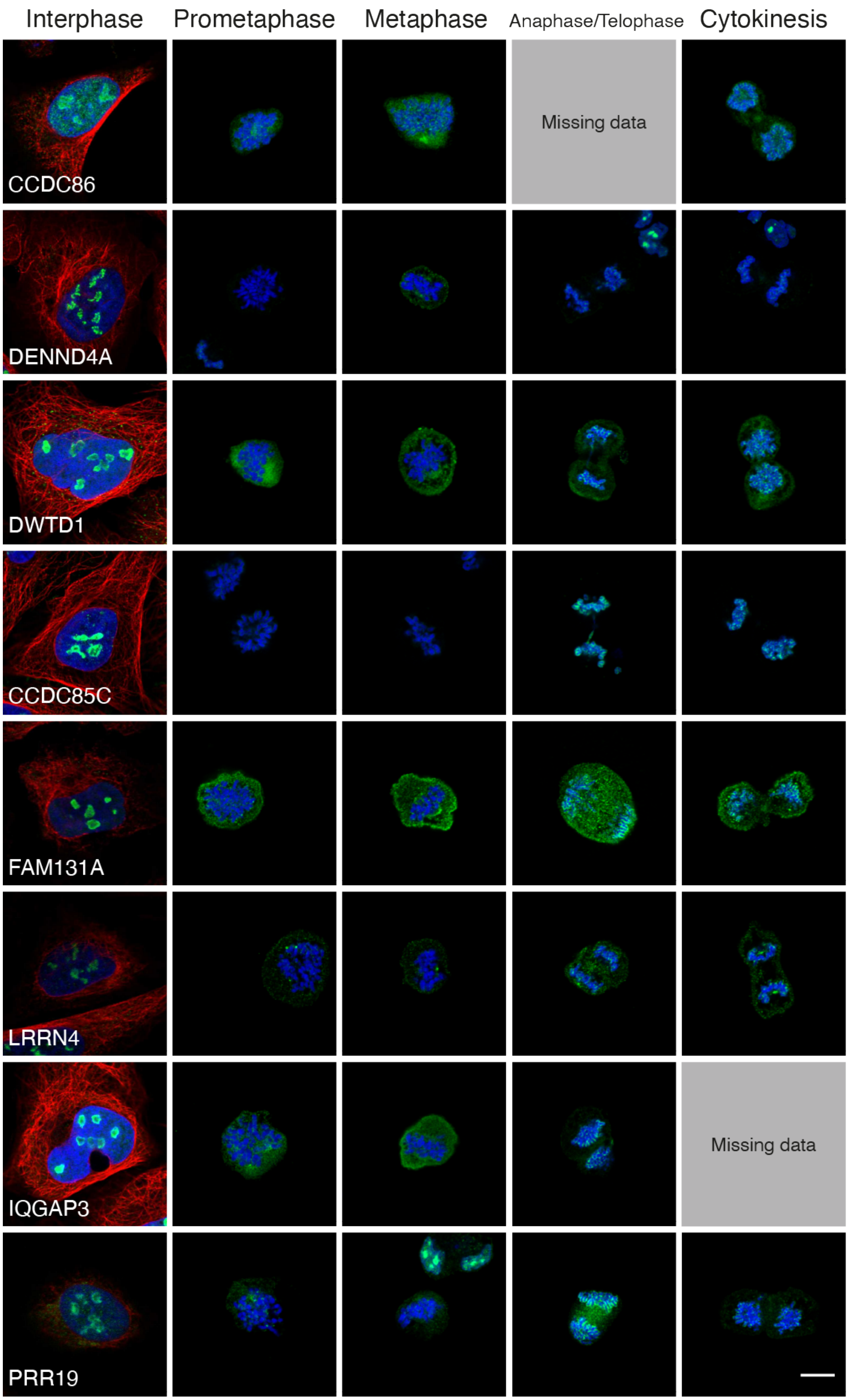

Interphase
Prometaphase
Metaphase
Anaphase/Telophase
Cytokinesis
CCDC86
Missing data
DENND4A
DWTD1
CCDC85C
FAM131A
LRRN4
IQGAP3
Missing data
PRR19

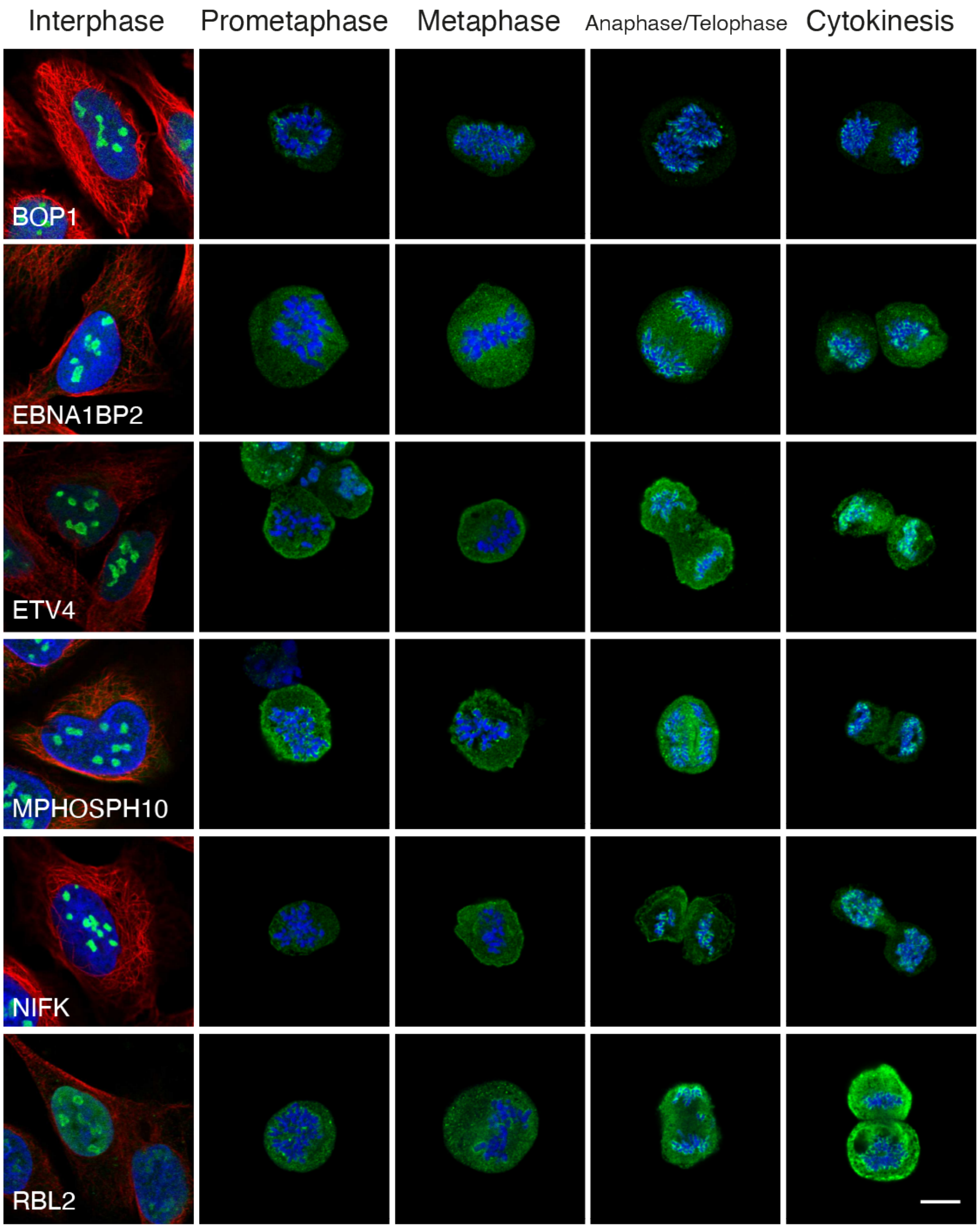

Appendix Figure S12

IF stainings of the proteins showing late recruitment to mitotic chromosomes. Protein of interest is shown in green, microtubules in red and DAPI in blue. Images of interphase cells were acquired from a different experiment and staining intensities cannot be compared between interphase and mitotic cells. For antibodies used, see Dataset EV3. Scale bar 10 μm.

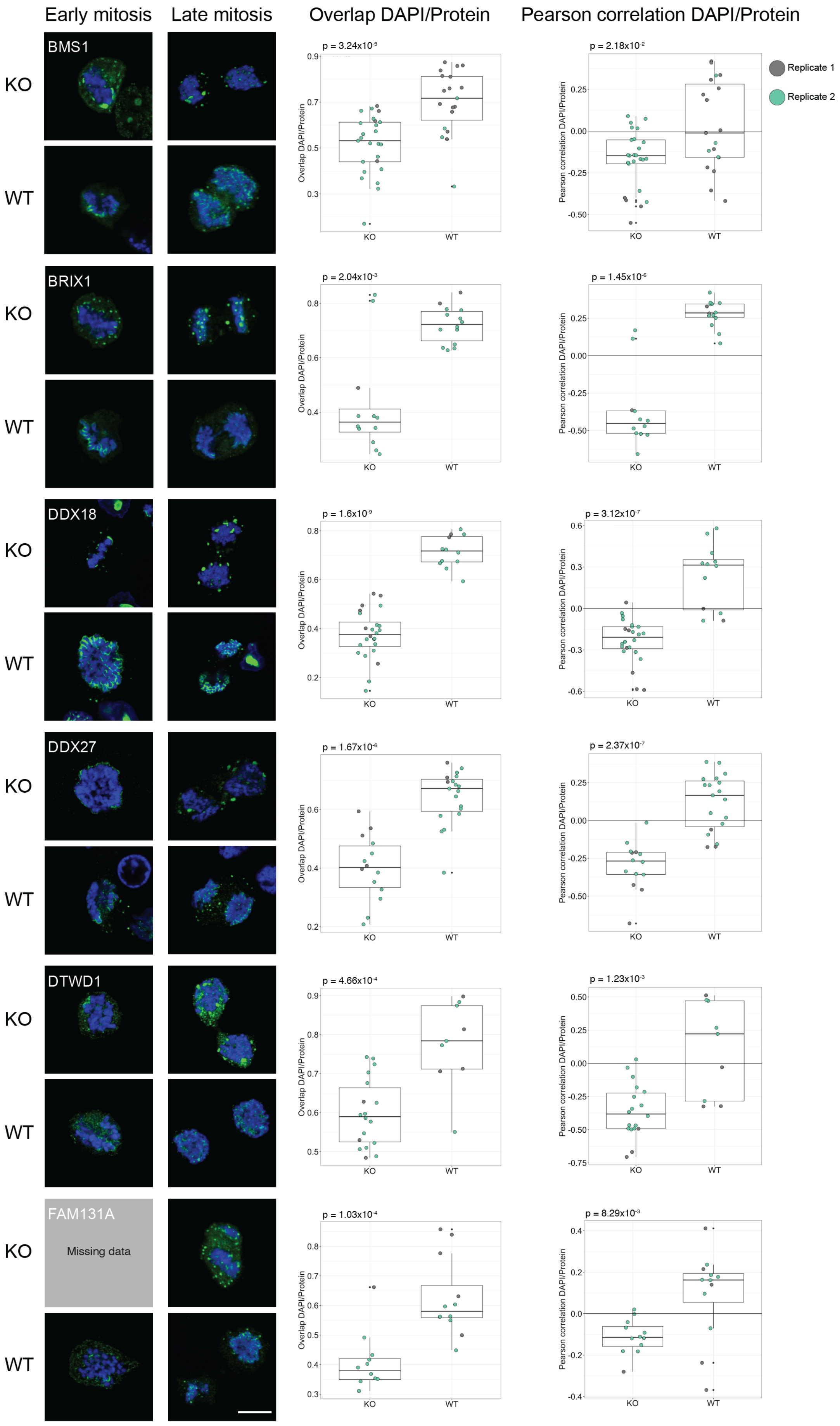

Early mitosis
Late mitosis
Overlap DAPI/Protein
Pearson correlation DAPI/Protein
Replicate 1
Replicate 2
BMS1
BRIX1
DDX18
DDX27
DTWD1
FAM131A
Missing data
KO
WT
p = 3.24x10-5
p = 2.18x10-2
p = 2.04x10-3
p = 1.45x10-6
p = 1.6x10-9
p = 3.12x10-7
p = 1.67x10-6
p = 2.37x10-7
p = 4.66x10-4
p = 1.23x10-3
p = 1.03x10-4
p = 8.29x10-3
Overlap DAPI/Protein
Pearson correlation DAPI/Protein

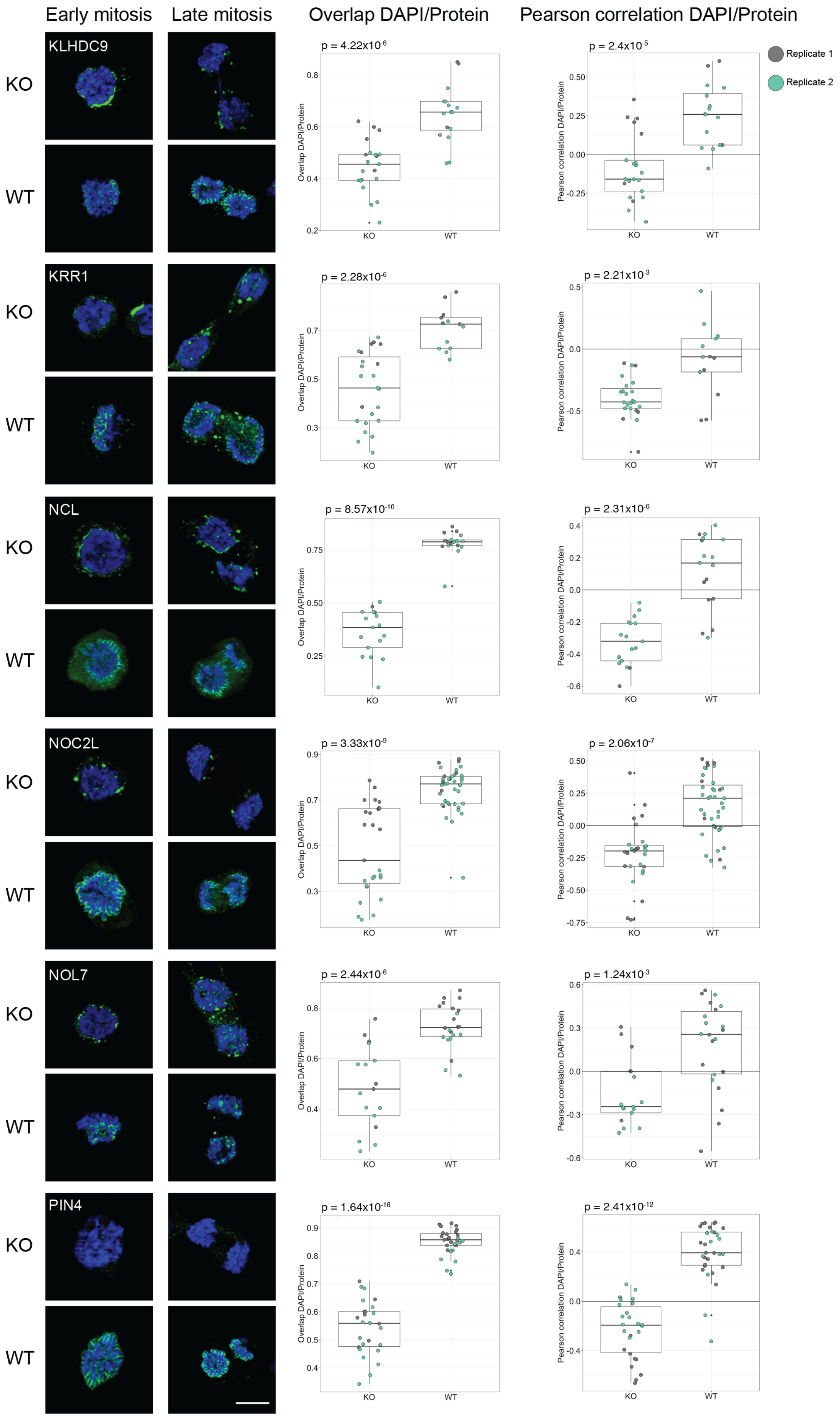
Early mitosis
Late mitosis
Overlap DAPI/Protein
Pearson correlation DAPI/Protein
Replicate 1
Replicate 2
KLHDC9
KRR1
NCL
NOC2L
NOL7
PIN4
KO
WT
p = 4.22x10-6
p = 2.4x10-5
p = 2.28x10-6
p = 2.21x10-3
p = 8.57x10-10
p = 2.31x10-6
p = 3.33x10-9
p = 2.06x10-7
p = 2.44x10-6
p = 1.24x10-3
p = 1.64x10-16
p = 2.41x10-12
Overlap DAPI/Protein
Pearson correlation DAPI/Protein

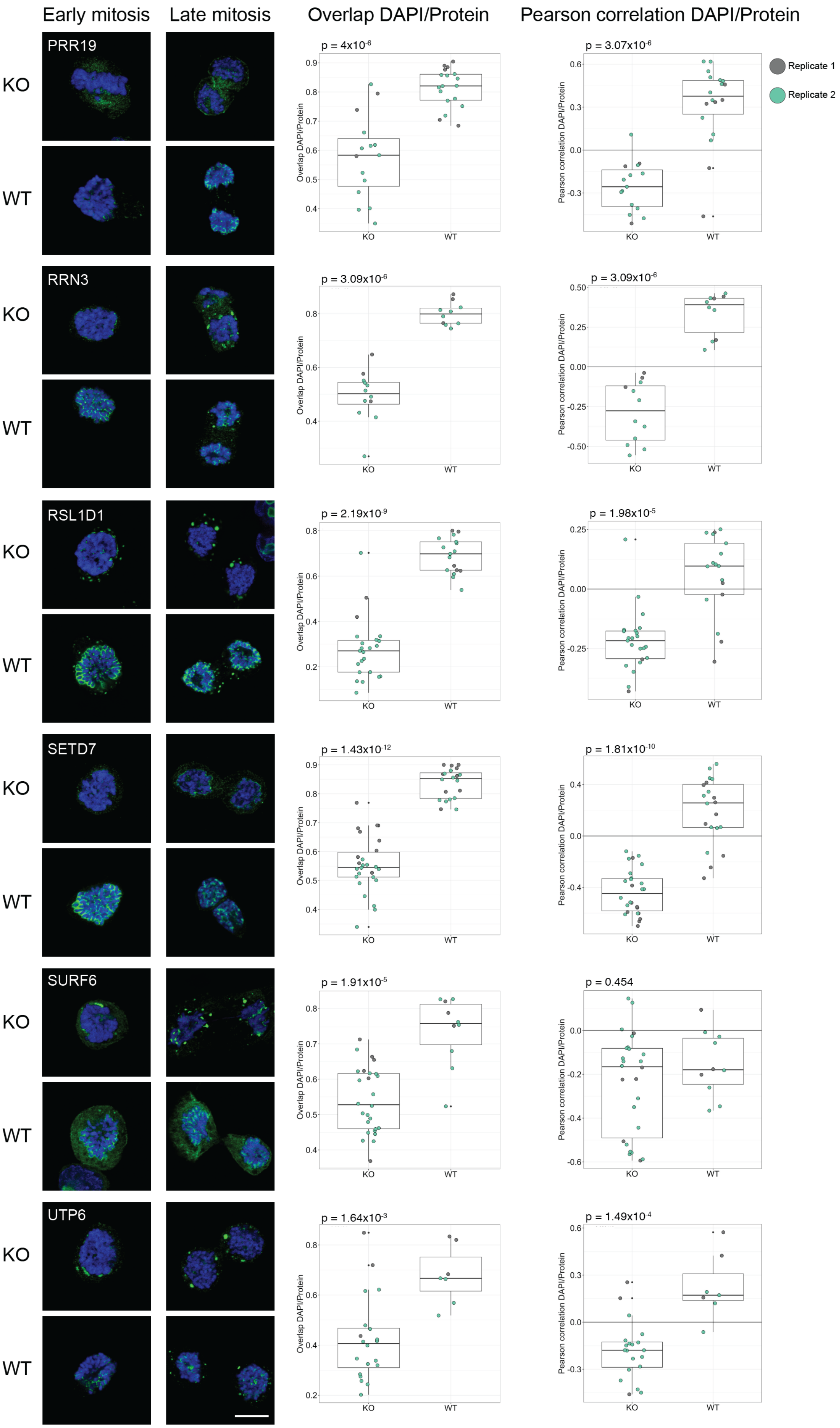
Early mitosis
Late mitosis
Overlap DAPI/Protein
Pearson correlation DAPI/Protein
Replicate 1
Replicate 2
PRR19
KO
WT
p = 4x10^-6
p = 3.07x10^-6
RRN3
KO
WT
p = 3.09x10^-6
p = 3.09x10^-6
RSL1D1
KO
WT
p = 2.19x10^-9
p = 1.98x10^-5
SETD7
KO
WT
p = 1.43x10^-12
p = 1.81x10^-10
SURF6
KO
WT
p = 1.91x10^-5
p = 0.454
UTP6
KO
WT
p = 1.64x10^-3
p = 1.49x10^-4

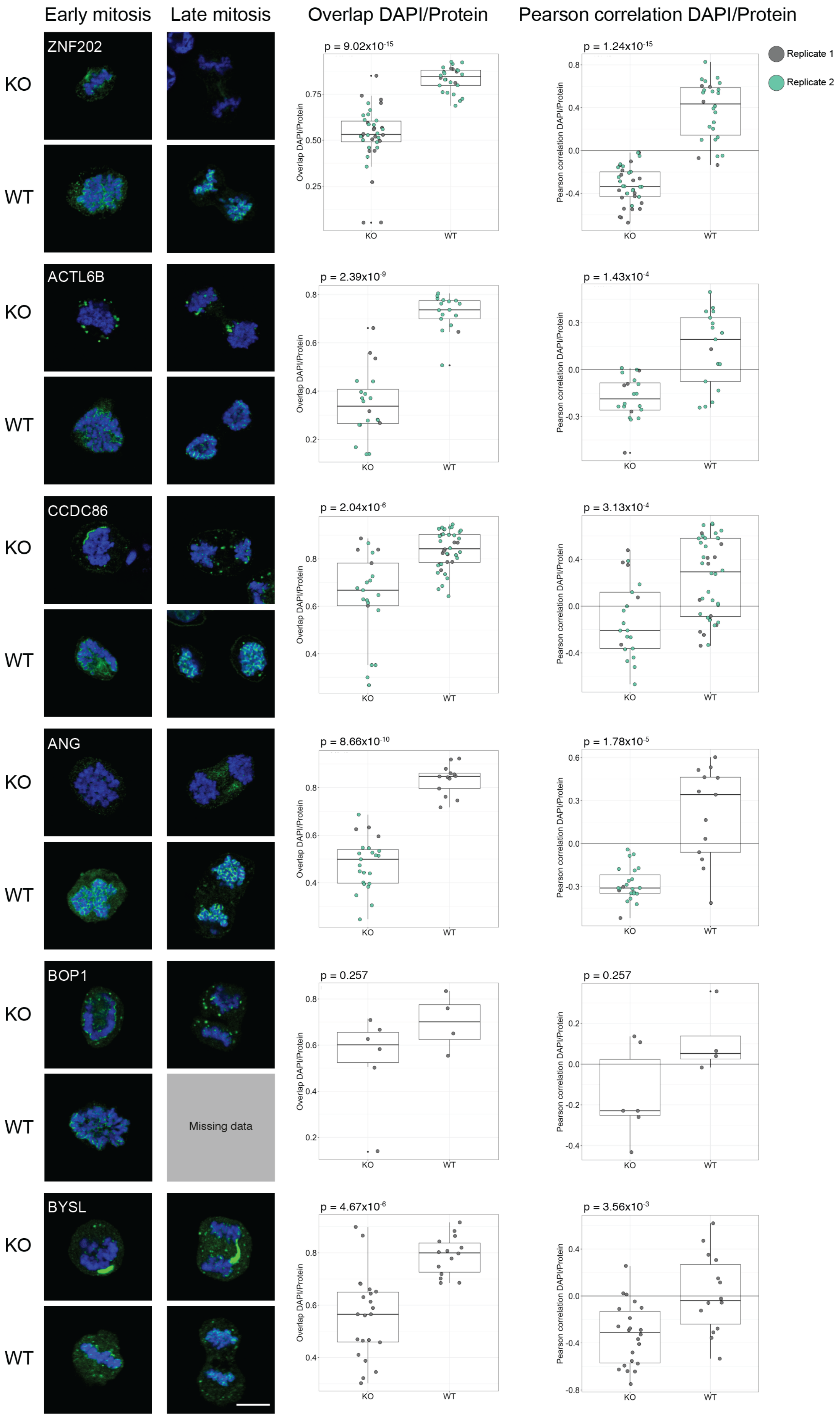
Early mitosis
Late mitosis
Overlap DAPI/Protein
Pearson correlation DAPI/Protein
Replicate 1
Replicate 2
ZNF202
KO
WT
p = 9.02x10^-15
p = 1.24x10^-15
ACTL6B
KO
WT
p = 2.39x10^-9
p = 1.43x10^-4
CCDC86
KO
WT
p = 2.04x10^-6
p = 3.13x10^-4
ANG
KO
WT
p = 8.66x10^-10
p = 1.78x10^-5
BOP1
KO
WT
Missing data
p = 0.257
p = 0.257
BYSL
KO
WT
p = 4.67x10^-6
p = 3.56x10^-3
Overlap DAPI/Protein
Pearson correlation DAPI/Protein

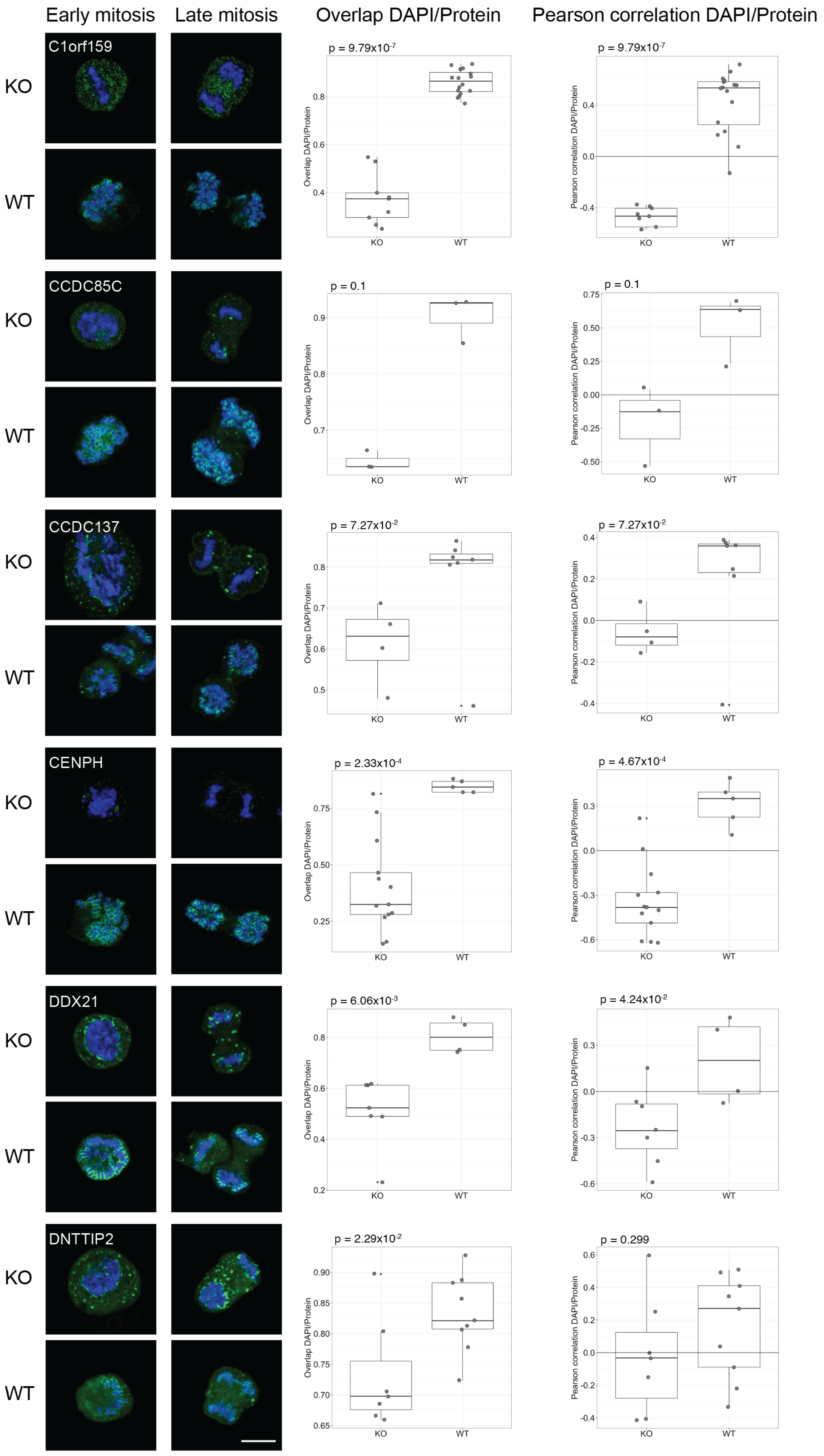
Early mitosis
Late mitosis
Overlap DAPI/Protein
Pearson correlation DAPI/Protein
C1orf159
CCDC85C
CCDC137
CENPH
DDX21
DNTTIP2
KO
WT
p = 9.79x10-7
p = 9.79x10-7
p = 0.1
p = 0.1
p = 7.27x10-2
p = 7.27x10-2
p = 2.33x10-4
p = 4.67x10-4
p = 6.06x10-3
p = 4.24x10-2
p = 2.29x10-2
p = 0.299
Overlap DAPI/Protein
Pearson correlation DAPI/Protein

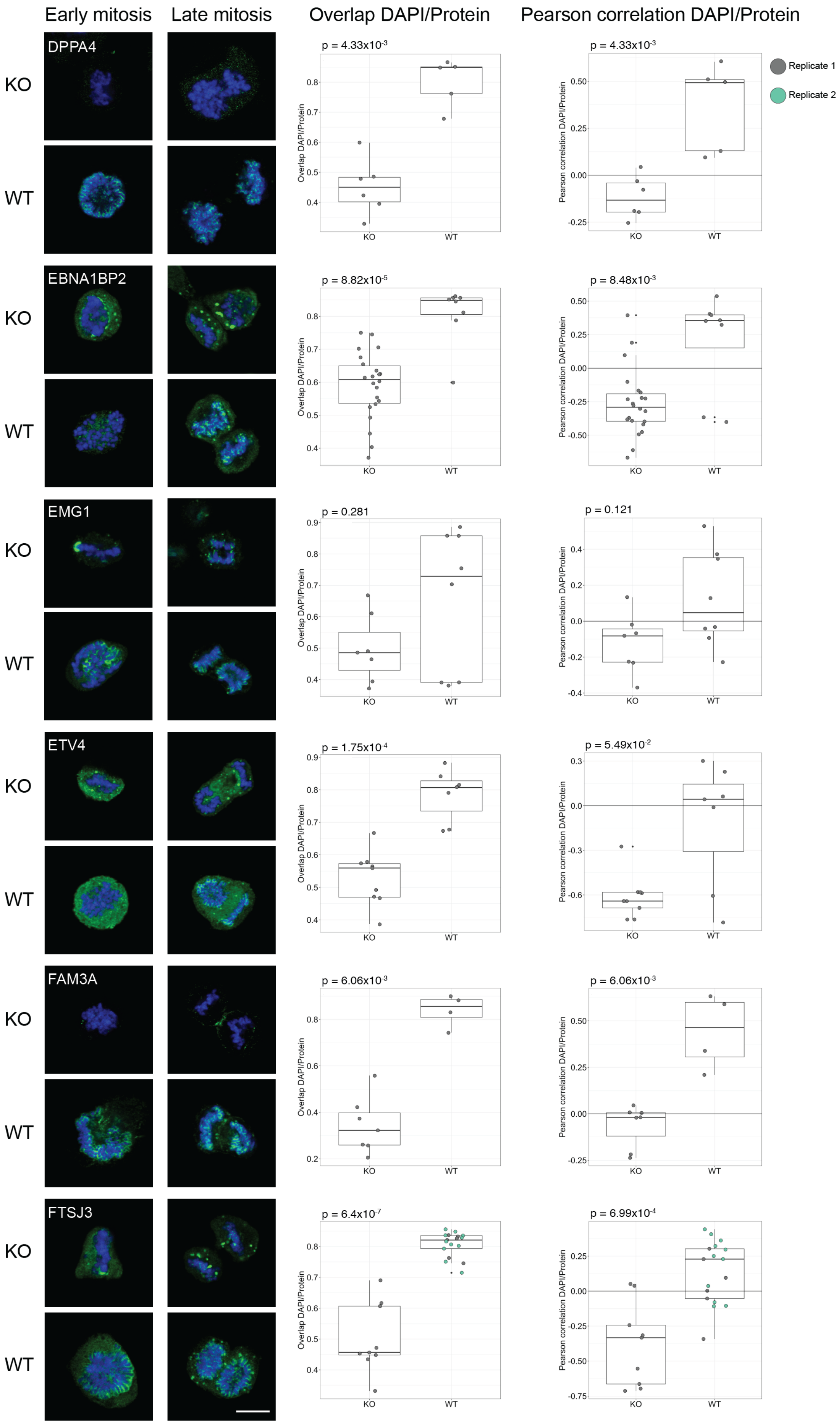
Early mitosis
Late mitosis
Overlap DAPI/Protein
Pearson correlation DAPI/Protein
Replicate 1
Replicate 2
DPPA4
EBNA1BP2
EMG1
ETV4
FAM3A
FTSJ3
KO
WT
Overlap DAPI/Protein
Pearson correlation DAPI/Protein
p = 4.33x10-3
p = 4.33x10-3
p = 8.82x10-5
p = 8.48x10-3
p = 0.281
p = 0.121
p = 1.75x10-4
p = 5.49x10-2
p = 6.06x10-3
p = 6.06x10-3
p = 6.4x10-7
p = 6.99x10-4

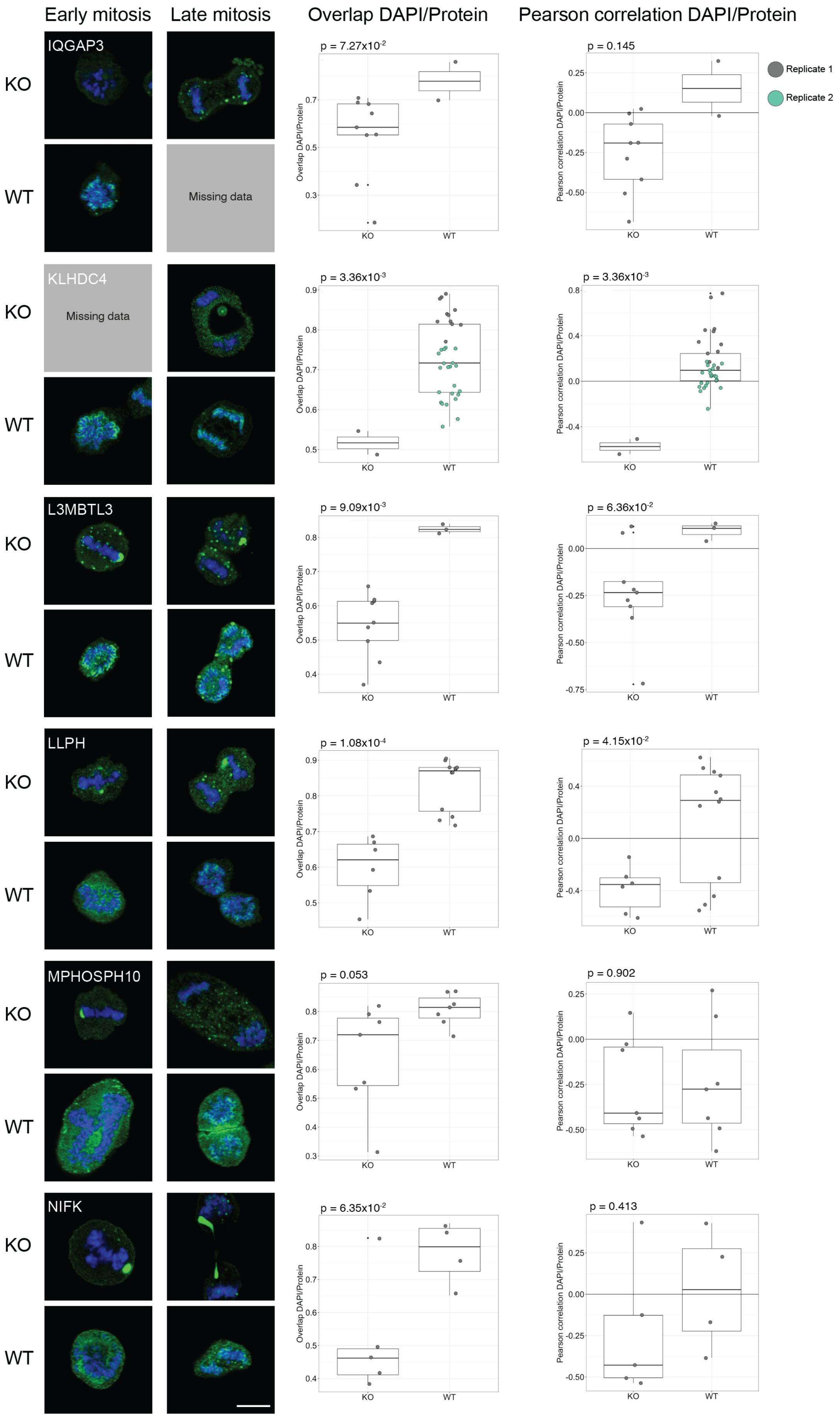
Early mitosis
Late mitosis
Overlap DAPI/Protein
Pearson correlation DAPI/Protein
Replicate 1
Replicate 2
IQGAP3
KO
WT
Missing data
p = 7.27x10-2
p = 0.145
KLHDC4
KO
WT
Missing data
p = 3.36x10-3
p = 3.36x10-3
L3MBTL3
KO
WT
p = 9.09x10-3
p = 6.36x10-2
LLPH
KO
WT
p = 1.08x10-4
p = 4.15x10-2
MPHOSPH10
KO
WT
p = 0.053
p = 0.902
NIFK
KO
WT
p = 6.35x10-2
p = 0.413
Overlap DAPI/Protein
Pearson correlation DAPI/Protein

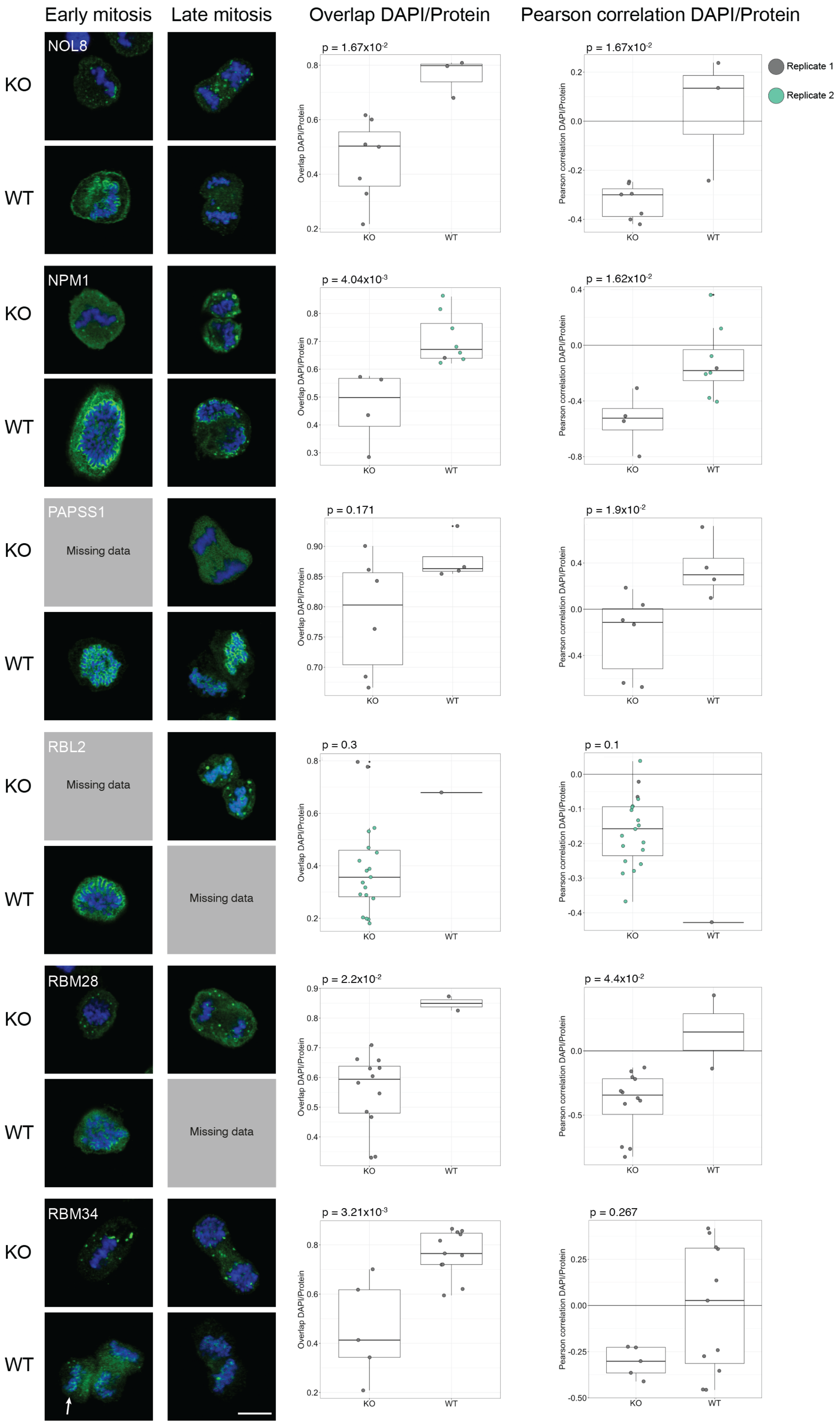

Early mitosis
Late mitosis
Overlap DAPI/Protein
Pearson correlation DAPI/Protein
Replicate 1
Replicate 2
NOL8
p = 1.67x10-2
p = 1.67x10-2
NPM1
p = 4.04x10-3
p = 1.62x10-2
PAPSS1
Missing data
p = 0.171
p = 1.9x10-2
RBL2
Missing data
Missing data
p = 0.3
p = 0.1
RBM28
Missing data
p = 2.2x10-2
p = 4.4x10-2
RBM34
p = 3.21x10-3
p = 0.267
KO
WT

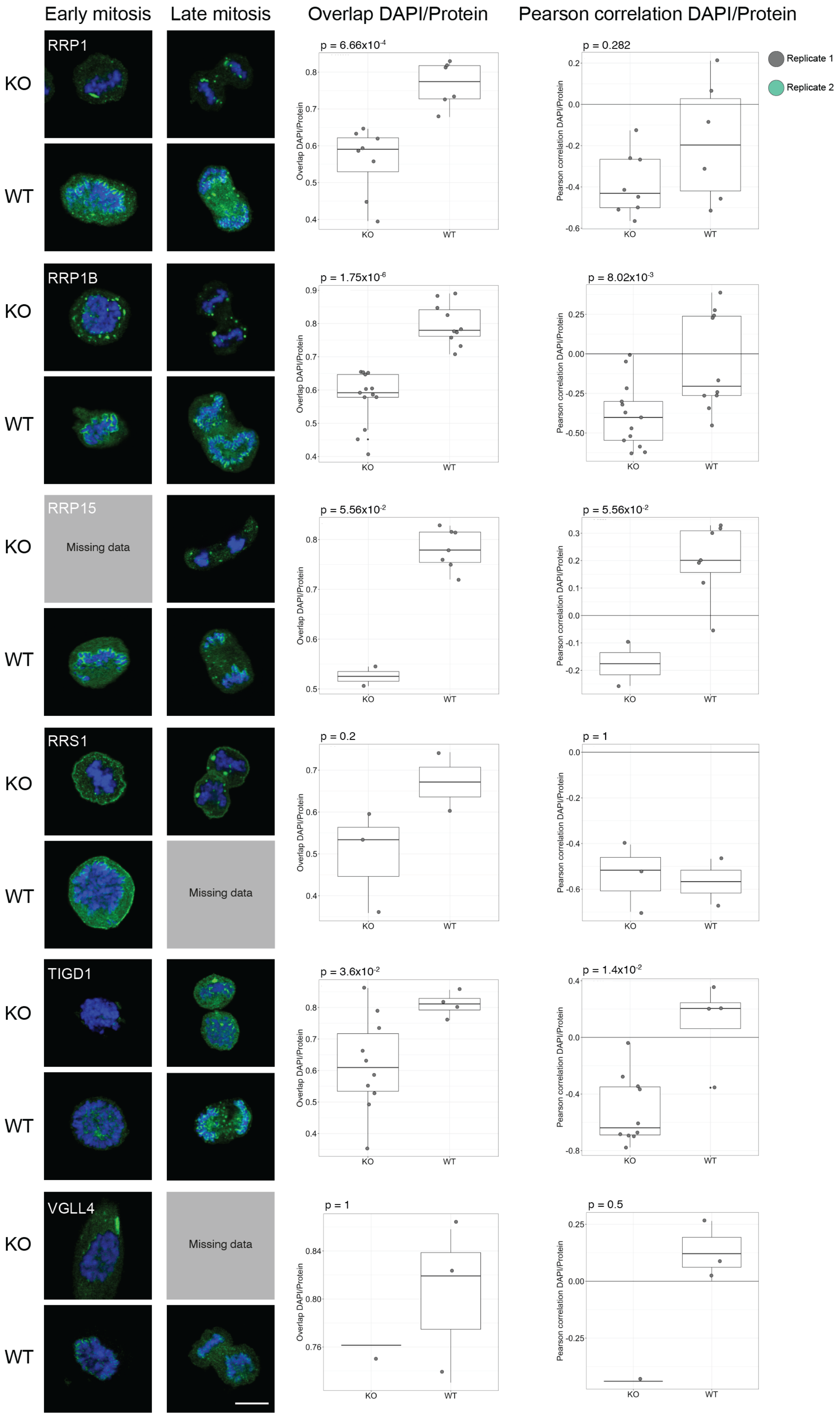

Early mitosis
Late mitosis
Overlap DAPI/Protein
Pearson correlation DAPI/Protein
Replicate 1
Replicate 2
RRP1
KO
WT
p = 6.66x10-4
p = 0.282
RRP1B
p = 1.75x10-6
p = 8.02x10-3
RRP15
Missing data
p = 5.56x10-2
p = 5.56x10-2
RRS1
Missing data
p = 0.2
p = 1
TIGD1
p = 3.6x10-2
p = 1.4x10-2
VGLL4
Missing data
p = 1
p = 0.5
Overlap DAPI/Protein
Pearson correlation DAPI/Protein

Appendix Figure S13

IF stainings of the mitotic chromosome proteins in WT HeLa and MKI67 KO HeLa cells. Protein is shown in green and DAPI in blue. For antibodies used, see Dataset EV3. Scale bar 10 µm. Box plots show measured overlap and Pearson correlation between the DAPI and protein staining (replicate 1 in grey and replicate 2 in green). A two-sided unpaired Wilcoxon test was applied to calculate the statistical significance between the phenotypes and the output p-value is stated in each plot.
